# Killer whales (*Orcinus orca*) in Iceland show weak genetic structure among diverse isotopic signatures and observed movement patterns

**DOI:** 10.1002/ece3.4646

**Published:** 2018-11-14

**Authors:** Sara B. Tavares, Filipa I. P. Samarra, Sonia Pascoal, Jeff A. Graves, Patrick J. O. Miller

**Affiliations:** ^1^ Sea Mammal Research Unit, Scottish Oceans Institute University of St Andrews St Andrews, Fife UK; ^2^ Marine and Freshwater Research Institute Reykjavík Iceland; ^3^ Department of Zoology University of Cambridge Cambridge UK; ^4^ Scottish Oceans Institute University of St Andrews St Andrews, Fife UK

**Keywords:** ecological niche, genetic differentiation, killer whales, microsatellites, *Orcinus orca*, population ecology

## Abstract

Local adaption through ecological niche specialization can lead to genetic structure between and within populations. In the Northeast Pacific, killer whales (*Orcinus orca*) of the same population have uniform specialized diets that are non‐overlapping with other sympatric, genetically divergent, and socially isolated killer whale ecotypes. However, killer whales in Iceland show intrapopulation variation of isotopic niches and observed movement patterns: some individuals appear to specialize on herring and follow it year‐round while others feed upon herring only seasonally or opportunistically. We investigated genetic differentiation among Icelandic killer whales with different isotopic signatures and observed movement patterns. This information is key for management and conservation purposes but also for better understanding how niche specialization drives genetic differentiation. Photo‐identified individuals (*N* = 61) were genotyped for 22 microsatellites and a 611 bp portion of the mitochondrial DNA (mtDNA) control region. Photo‐identification of individuals allowed linkage of genetic data to existing data on individual isotopic niche, observed movement patterns, and social associations. Population subdivision into three genetic units was supported by a discriminant analysis of principal components (DAPC). Genetic clustering corresponded to the distribution of isotopic signatures, mtDNA haplotypes, and observed movement patterns, but genetic units were not socially segregated. Genetic differentiation was weak (*F*_ST_ < 0.1), suggesting ongoing gene flow or recent separation of the genetic units. Our results show that killer whales in Iceland are not as genetically differentiated, ecologically discrete, or socially isolated as the Northeast Pacific prey‐specialized killer whales. If any process of ecological divergence and niche specialization is taking place among killer whales in Iceland, it is likely at a very early stage and has not led to the patterns observed in the Northeast Pacific.

## 1 INTRODUCTION

The socioecological characteristics of a species, such as fidelity to specific natal breeding or feeding grounds (e.g., Carroll et al., 2015; Kershaw et al., 2017; Valenzuela, Sironi, Rowntree, & Seger, 2009) and local adaptation to specific habitats, for example through niche specialization (e.g., Foote et al., 2016; Hoelzel, Dahlheim, & Stern, 1998; Smith & Skúlason, 1996), can determine genetic divergence of populations. Additionally, socioecological characteristics can affect patterns of mating and dispersal within populations creating fine‐scale genetic structure (e.g., Archie et al., 2008; Garant, Dodson, & Bernatchez, 2000; Storz, 1999). Ultimately, these may lead to speciation (Foote et al., 2016; Smith & Skúlason, 1996; Storz, 1999). Understanding patterns of population genetic structure and the processes driving those patterns is highly relevant for conservation purposes, since smaller population units are more vulnerable to extinction (Stevick et al., 2006).

The killer whale (*Orcinus orca*) is an apex predator widely distributed throughout all oceans (Forney & Wade, 2006). Much knowledge about killer whales comes from long‐term studies on two sympatric and socially segregated ecotypes in the Northeast Pacific (e.g., Ford et al., 1998; Ford, Ellis, & Balcomb, 2000): (a) the “resident” fish‐eating (hereafter termed residents) killer whales, which feed primarily on salmon; and (b) the mammal‐eating (also referred to as “transients” or Bigg's killer whales), which feed on marine mammals. Between the two ecotypes, there is strong genetic differentiation (Barrett‐Lennard, 2000; Foote et al., 2016; Hoelzel et al., 1998, 2007, 2002; Morin et al., 2010, 2015 ; Moura et al., 2015; Moura, Kenny, et al., 2014; Parsons et al., 2013). At the ecotype and subpopulation level, there are high levels of philopatry, with no dispersal of either sex, thought to promote stable foraging traditions by knowledge transfer within matrilineal units, acting to buffer kin fitness (Barrett‐Lennard, 2000; Brent et al., 2015; Foster et al., 2012; Riesch, Barrett‐Lennard, Ellis, Ford, & Deecke, 2012).

Contrarily to the Northeast Pacific, although dietary variation and some degree of ecological divergence have been reported among North Atlantic killer whales (Foote et al., 2013, 2011; Foote, Newton, Piertney, Willerslev, & Gilbert, 2009; Foote, Vester, Víkingsson, & Newton, 2012), the link between genetic divergence and resource specialization is less clear. In Iceland, not all killer whales appear to specialize on herring and follow it year‐round. Photo‐identification of killer whales in Icelandic herring overwintering‐ and summer‐spawning grounds showed that some individuals are sighted in both summer and winter seasons but others are seen only seasonally (Samarra, Tavares, et al., 2017). Also, some individuals observed only in the winter season were seen moving to Scotland in the summer, where they were seen feeding upon marine mammals (Samarra & Foote, 2015). Isotopic analyses of biopsy sampled individuals with different observed movement patterns show that these largely correspond to different isotopic niche widths (Samarra, Vighi, Aguilar, & Víkingsson, 2017). Individuals seen in both seasons had lower nitrogen stable isotope ratios (^15^N/^14^N, represented as δ^15^N), consistent with a diet predominantly composed of herring, while killer whales only seen seasonally (including sampled individuals that travel between Iceland and Scotland) exhibited larger variation in δ^15^N, suggesting that some individuals have a diet including other prey (Samarra, Vighi, et al., 2017). However, there is no social isolation between individuals with different observed movement patterns and isotopic signatures, that is, putative herring‐specialists remaining year‐round in Iceland have been photographed in close proximity with Icelandic‐Scottish killer whales (Tavares, Samarra, & Miller, 2017). It is unknown whether the apparent absence of social isolation in the Icelandic population corresponds to an absence of genetic divergence among individuals with different isotopic values and observed movement patterns.

The aim of our study was to investigate patterns of genetic structure among Icelandic killer whales. Tissue samples of photo‐identified individuals allowed for the correlation of genetic data with existing data of photographic mark‐recapture and individual isotopic niche. Population subdivision based on microsatellite markers was estimated. Genetic diversity, mitochondrial DNA (mtDNA) haplotype frequency and individual data on observed movement patterns, social associations, and isotopic signatures were used to measure the divergence of putative genetic units. We interpret the identified genetic patterns among Icelandic killer whales, discuss potential ecological and behavioral processes driving them and how they correspond to the patterns of the best‐studied Northeast Pacific ecotypes.

## 2 MATERIALS AND METHODS

### 2.1 Sample and data collection

**Figure 1:**
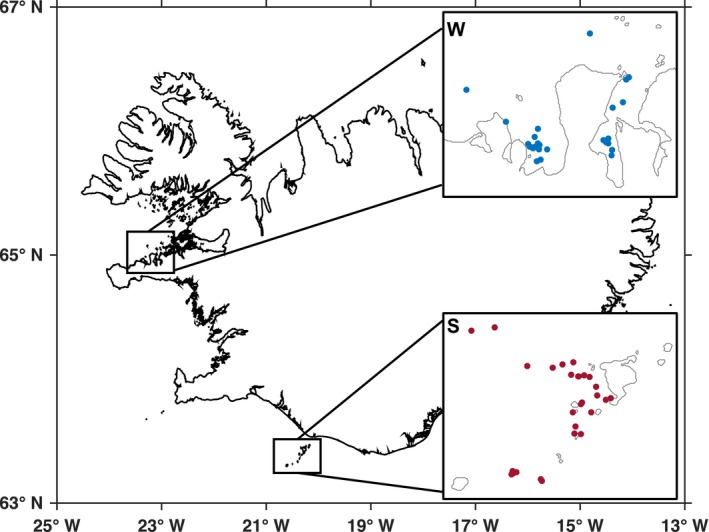
Locations of biopsy skin samples collected in Iceland from killer whales in Grundarfjörður and Kolgrafafjörður in the winter (W) and Vestmannaeyjar in the summer (S)

Biopsy samples of wild Icelandic killer whales (*N* = 60) were collected from a research vessel in both herring overwintering‐ (winter) and spawning‐ (summer) grounds where killer whales can be seasonally found, coinciding with seasonal herring migration into those locations. In the winter, samples were collected off Grundarfjörður and Kolgrafafjörður (Figure 1, W; West Iceland) in February and March 2013 and 2014. In the summer, samples were collected in July 2014 and 2015 off Vestmannaeyjar (Figure 1, S; South Iceland). Skin and blubber samples were collected from photo‐identified individuals using a Remington rolling block system rifle (“Larsen” long‐range biopsy system) with 35 or 40 mm sterilized biopsy tips in 2013 and an ARTS pneumatic darting system (Kvadsheim, Lam, Miller, Alves, & Others, 2009) with stainless steel 25 mm sterilized biopsy tips in subsequent years. In general, biopsy samples were collected from the mid‐lateral region of the body, below the dorsal fin. Skin samples, used in subsequent analyses, were stored in 70% ethanol at −20 °C. Only adults or subadult individuals were sampled. One additional skin sample was collected from a necropsy performed by the Marine and Freshwater Research Institute (Iceland) in March 2016 on a photo‐identified killer whale stranded near Grundarfjörður. All field research and sample collection, designed for minimum distress to the animals, were approved by the School of Biology Ethics Committee of the University of St Andrews and carried out in compliance with local regulations and under permits provided by the Ministry of Fisheries (institutional permit for the Marine and Freshwater Research Institute, Reykjavík).

### 2.2 DNA extraction and genetic sex identification

Total genomic DNA (*N* = 61) was extracted from skin samples using standard proteinase K digestion and phenol/chloroform methods (Sambrook, Fritsch, & Maniatis, 1989) modified for small samples by Baker et al. (1994). DNA was quantified on a NanoDrop ND‐1000 Spectrophotometer and standardized to 10–20 ng/µl. The sex of individual whales was genetically identified using the protocol of Jayasankar, Anoop, and Rajagopalan (2008) which amplifies a 210–224 base pair (bp) fragment of the Y‐chromosome‐specific region (SRY) in males and a 442–445 bp size fragment of the ZFX/ZFY region in both sexes. Polymerase chain reaction (PCR) products were run on agarose gel stained with ethidium bromide (EtBr) and visualized under UV light.

### 2.3 mtDNA control region haplotype identification

Two sections of the 5′ end of the mtDNA control region were amplified as in Foote et al. (2009): a longer fragment (about 480 bp in length) using primers H16498 (5′‐CCT GAA GTA AGA ACC AGA TG‐3′) and L15812 (5′‐CCT CCC TAA GAC TCA AGG AAG‐3′) (Zerbini et al., 2007), and an additional non‐overlapping smaller fragment (about 131 bp size) using primers DH6 (5′‐AAA TAC AYA CAG GYC CAG CTA‐3′) and DL5 (5′‐CCY CTT AAA TAA GAC ATC TCG‐ ATG G‐3′) (Morin et al., 2006). For the longer fragment, each 20 μl of PCR contained 1 μl of 10–20 ng of extracted DNA, 1× PCR buffer, 1.5 mM MgCl_2_ (magnesium chloride), 0.2 μM of each primer, 0.2 mM of mixed dNTPs (i.e., deoxyribonucleotide triphosphates, referring to the four different dNTPs: dATP, dCTP, dGTP, and dTTP), and 0.1 μl of AmpliTaq® DNA Polymerase (Applied Biosystems, Foster City, CA, USA). For the smaller fragment, the quantities were the same except for the primers, which were increased to 0.3 μM of each. PCR amplifications were performed using a G‐Storm GS1 thermal cycler (Gene Technologies) with an initial denaturation step at 95°C for 2 min, followed by a specific number of cycles (33 cycles for the longer fragments and 38 cycles for the smaller fragments) of denaturation at 95°C for 15 s, annealing for either 30 s at 54°C for the longer fragment or for 1 min at 57°C for the smaller fragment, and extension at 72°C for 1 min, followed by a final extension at 72°C for 5 min. Successful amplification was confirmed using agarose gel, EtBr staining, and UV visualization. Negative controls without DNA were included in all PCR plates to monitor for contamination during the PCR set up. Excess dNTPs and unincorporated primers from the completed amplifications were removed using either Illustra ExoProStar 1‐Step (GE Healthcare Life Sciences) or ExoSAP‐IT™ PCR Product Cleanup Reagent (Thermo Fisher Scientific Inc.).

PCR products were sequenced in both directions on an ABI 3730 DNA sequencer (Applied Biosystems) at Edinburgh Genomics (University of Edinburgh). Seven randomly selected samples (>10% of the dataset) were re‐sequenced to ensure consistency. All sequences were visually inspected using the software FinchTV v1.4.0 (Geospiza, Inc., Seattle, WA) by two of the authors independently: forward and reverse readings of the same sample were compared and any inconsistency was corrected according to the result from the clearest sequence. For each sample, the sequences of the small and longer fragments were concatenated. To determine individual mtDNA haplotype, the final sequences were aligned against 11 previously published sequences for the same genetic regions in North Atlantic killer whales (Foote et al., 2009; Hoelzel et al., 2002) using Clustal W multiple alignment method (Thompson, Higgins, & Gibson, 1994) as implemented in the software BioEdit v7.2.5 (Hall, 1999).

### 2.4 Microsatellite genotyping

Twenty‐two loci were selected from the literature (Appendix S1). Fifteen of these loci were previously used by Foote et al. (2011) for genetic differentiation of North Atlantic killer whales and seven by Parsons et al. (2013) for genetic differentiation among northern North Pacific killer whales. The microsatellites were arranged in five groups for multiplex PCR, according to the expected size range of each marker, the dye color used, and the annealing temperature in optimum PCR conditions (Appendix S1). We used QIAGEN**® **Multiplex PCR kits to amplify the loci with the fluorescent M13 tail single‐reaction nested PCR method (Schuelke, 2000) with four different color‐specific tails (Tysklind, 2009). Multiplex PCR reactions of 10 μl contained 5 μl of 2× QIAGEN Multiplex PCR Master Mix, 0.4 μl of 0.5 μM forward primer mix (i.e., the forward primers of each multiplex group, each with a different tail in the 5′ depending on the color label), 0.4 μl of 5 μM reverse primer mix (i.e., the reverse primers of each multiplex group), 0.4 μl of 5 μM mix of four different labelled primers (FAM, NED, VIC and PET), 2 μl of RNase‐free water, and 1–2 μl of 10–20 ng of extracted DNA. DNA was amplified on a G‐Storm GS1 thermal cycler (Gene Technologies) with an initial 15 min step at 95°C, 13 cycles of denaturation at 94°C for 30 s, annealing for 90 s at 60°C for groups I–IV and 55°C for group V (Appendix S1), and extension at 72°C for 60 s, followed by 31 cycles of denaturation at 94°C for 30 s, annealing at 50°C for 90 s and extension at 72°C for 60 s, followed by a final extension at 60°C for 30 min. Successful amplification was confirmed by agarose gel, EtBr staining, and UV visualization. All amplifications included a negative control to detect contamination. Fragment analysis was conducted on an ABI 3730 DNA sequencer (Applied Biosystems) at Edinburgh Genomics (University of Edinburgh) using LIZ 500 (Applied Biosystems) as internal standard. Seven samples (>10% of the dataset) selected at random were re‐amplified and re‐genotyped for all loci to ensure consistent allele sizing. Alleles were sized using Peak Scanner Software 2 (Applied Biosystems) by two of the authors independently.

The existence of matching genotypes in the dataset was investigated using CERVUS v3.0.7 (Kalinowski, Taper, & Marshall, 2007). Allele frequencies were calculated in GENALEX v6.503 (Peakall & Smouse, 2006). All loci were inspected for scoring errors or null alleles using MICRO‐CHECKER v2.2.3 (Van Oosterhout, Hutchinson, Wills, & Shipley, 2004). Deviations from Hardy–Weinberg equilibrium (HWE) and linkage equilibrium were tested using 1,000 iterations in GENEPOP v4.2 (Rousset, 2008). The analyses were conducted for the whole dataset and for each genetic unit identified by the clustering method (see Results section). Significance levels were corrected for multiple testing using the sequential Bonferroni correction (Holm, 1979) for these tests and all subsequent multiple comparisons in the study.

### 2.5 Genetic differentiation and population subdivision

To identify genetic structure in the microsatellite markers’ dataset, two different clustering methods were used. First, we used Bayesian clustering analysis for detection of genetically differentiated clusters (*K*) performed in STRUCTURE v2.3.4 (Pritchard, Stephens, & Donnelly, 2000). Ten independent runs for *K* values set from 1 to 10 were performed using a burn‐in period of 100,000 iterations followed by 1,000,000 Markov chain Monte Carlo steps. The admixture model with correlated allele frequencies, recommended when population structure is likely subtle (Falush, Stephens, & Pritchard, 2003), was chosen, and no a priori information on the origin of the sample was indicated. The mean log‐likelihood (L(*K*)) of each *K*, calculated in STRUCTURE HARVESTER v.0.6.94 (Earl & VonHoldt, 2012), was used as choice criterion to select likely number of *K* (Pritchard et al., 2000).

Secondly, we used a discriminant analyses of principal components method (DAPC) (Jombart, Devillard, & Balloux, 2010) in the package adegenet (Jombart, 2008) in R 3.4.3 (R Core Team, 2015). DAPC is a multivariate clustering method where individuals are clustered by genetic similarity not assuming any population genetic model, and efficiently detects genetic hierarchical structure (Jombart et al., 2010). We performed the DAPC analysis following the recommendations of Jombart and Collins (2015). Briefly, the most likely number of clusters was first assessed using a *K*‐means method setting the maximum number of clusters to 40 and retaining all principal components. The most likely number was defined by the lowest BIC (Bayesian Information Criterion) value and the elbow in the BIC curve. Then, the genetic data were transformed using Principal Component Analyses and a linear discriminant analysis was performed on the retained principal components (no more than 80% were retained to avoid over‐fitting). Each individual was assigned to a genetic unit according to its maximum membership probability. The robustness of the division was confirmed by rerunning DAPC after removing one individual from the pairs of individuals showing a relatedness coefficient superior or equal to 0.45 as in Rosel, Hansen, and Hohn (2009) and Louis et al. (2014); pairwise relatedness values were estimated within each putative genetic unit (as in Louis et al., 2014, to ensure allele frequencies are affected by the inferred genetic structure) in KINGROUP v2_101202 (Konovalov, Manning, & Henshaw, 2004) using Queller and Goodnight's (1989) relatedness estimator in its symmetric form (Goodnight & Queller, 1999).

To characterize the level of differentiation of putative genetic units, we calculated pairwise and overall *F*_ST_ values for microsatellite loci in FSTAT v2.9.3.2 (Goudet, 2001) using the computational methods of Weir and Cockerham (1984). The level of significance for pairwise *F*_ST_ values was assessed using 3,000 permutations and analyses were also performed with the dataset excluding closely related individuals. A randomization procedure in R (R Core Team, 2015) was used to test the null hypothesis that pairwise *F*_ST_ values obtained are no different than expected by comparing sets of randomly selected individuals. First, we created a distribution of *F*_ST_ values for each combination of pairs of genetic units by calculating pairwise *F*_ST_ values with 10 randomly sampled individuals from each genetic unit and repeating this procedure 1,000 times. Then, we created a range of *F*_ST_ values by calculating pairwise *F*_ST_ values from two random pools each with 10 randomly sampled individuals from the whole dataset, repeating this procedure 1,000 times. Finally, we tested whether the distribution of pairwise *F*_ST_ values of the pairs of genetic units were significantly different from pairwise *F*_ST_ values of the random groups.

Microsatellite loci diversity indices were calculated for each putative genetic unit (and also per locus): the mean sample size per locus (*n*), the mean number of alleles per locus (*k*) and the observed and expected heterozygosities (*H*_o_ and *H*_e_, respectively) in CERVUS (Kalinowski et al., 2007); the number of private alleles (PA) in GENALEX (Peakall & Smouse, 2006); the mean allelic richness (AR, i.e., mean number of alleles per locus averaged over the number of loci and adjusted for sample size) and the inbreeding coefficient (*F*_IS_) in FSTAT (Goudet, 2001). Mean AR and *H*_o_ values were compared among genetic units using two‐sample Wilcoxon tests in R (R Core Team, 2015). The distribution of mtDNA haplotypes within each unit was assessed and the relationship between haplotype and genetic unit was tested using a 2x3 Fisher's exact test in R (R Core Team, 2015).

### 2.6 Genetic units and movement patterns, isotopic niche width and social segregation

The variation in movement patterns within each putative genetic unit was investigated by assigning an observed movement pattern to each sampled individual based on Samarra, Tavares, et al. (2017). Individuals were considered “seasonal” or “year‐round” if photographed in Iceland only seasonally (either in the winter or in the summer) or if sighted in both herring overwintering‐ and spawning‐grounds, respectively. We tested for significant differences in the ratio of seasonal versus year‐round individuals among genetic units using a 2×3 Fisher's exact test in R (R Core Team, 2015). Complete movement patterns of individuals and the extent of dispersal from Iceland are unknown. To date, based on sightings by the public and whale‐watching companies shared on social media, six of the sampled individuals only seen in Iceland in the winter have now been confirmed having traveled to Scotland in spring and summer (S.B. Tavares, pers. obs.).

For the majority of the sampled individuals (*N* = 56), stable isotope ratios analyzed from skin samples (specifically of nitrogen denoted as δ^15^N and carbon denoted as δ^13^C) were available from Samarra, Vighi, et al. (2017). Differences in the isotopic niche width between putative genetic units were assessed using the area of standard ellipses corrected for sample size (SEA_C_) and statistically tested by comparing the probability distributions of Bayesian estimates of SEA_C_ (2,000,000 iterations and 10,000 burn‐in iterations) with the SIAR (Parnell & Jackson, 2013) and SIBER packages (Jackson, Inger, Parnell, & Bearhop, 2011) in R (R Core Team, 2015).

Association data were available from Tavares et al. (2017) for sampled individuals with a minimum sighting total of five days (*N* = 47). Tavares et al. (2017) considered individuals associated in a day if photographed by the same camera/photographer within 20 s and used the half‐weight index (HWI, ranging from 0 to 1) to quantify associations between pairs of individuals. The existence and strength of social associations between individuals from different putative genetic units was used to evaluate the social isolation of genetic units. Differences in mean association within versus between putative genetic units were assessed by bootstrapping the individuals 10,000 times in R (R Core Team, 2015). Associations among individuals were visualized in a social network plotted using the package igraph (Csardi & Nepusz, 2006) in R (R Core Team., 2015).

### 2.7 Sex‐biased dispersal, recent migration rates, and changes in effective size

Sex‐bias in dispersal among putative genetic units was tested using the biased dispersal option in FSTAT (Goudet, 2001). Differences in sex‐specific *F*_ST_ and variance of assignment index (vAI) between males and females from different genetic units, were tested by generating null distributions with 10,000 permutations. Since only adults or sub‐adults were sampled, the whole dataset was used in this test. The mean number of successfully reproducing migrants per generation among movement patterns (*N*_m_) was estimated using GENEPOP (Rousset, 2008). Recent migration rates (i.e., past 1–3 generations) among putative genetic units were estimated using the Bayesian method implemented in BAYESASS v3.0.4 (Wilson & Rannala, 2003). As recommended by Rannala (2015), preliminary runs were performed in BAYESASS to assess convergence and mixing using Tracer v1.6.0 (Rambaut, Drummond, Xie, Baele, & Suchard, 2018), which showed that convergence was usually reached before 100,000,000 iterations. Therefore, we performed five different runs with 300,000,000 Markov chain Monte Carlo iterations, a burn‐in of 10,000,000 iterations, and a sampling interval of 2000 iterations. Consistency in the results and effective convergence was confirmed for all runs.

Recent change in effective size of the putative genetic units was tested using BOTTLENECK v1.2.02 (Piry, Luikart, & Cornuet, 1999) using two different mutation models of microsatellite evolution: the infinite allele model and the two‐phase model, which allows multiple‐step mutations. Parameters were set to 10,000 repetitions, with 70% single‐step mutations in the two‐phase model and a variance of 12 for multiple‐step mutations. Significance of results for both mutation models was assessed using the Wilcoxon test, which is more robust when used with few polymorphic loci (Piry et al., 1999).

## 3 RESULTS

### 3.1 Sample collection and DNA extraction

The 61 photo‐identified Icelandic killer whales were successfully genotyped and assigned a sex based on genetic analysis (18 females and 43 males): 31 individuals (nine females and 22 males) were seen in both seasonal herring grounds and the remainder of the individuals were seen only seasonally (11 were only seen in the winter, six were seen only in the winter and traveled to Scotland in the spring and summer, and 13 were only seen in the summer).

### 3.2 mtDNA control region haplotype identification

Sequences of the control region (~611 bp) were generated for all individuals and two different haplotypes were identified, haplotypes 33 (published by Hoelzel et al., 2002) and 34 (published by Foote et al., 2009). The two haplotypes varied in only one site which was positioned in the longer fragment amplified in the mtDNA control region, with no variation seen in the smaller fragment. Genetic differentiation based on mtDNA was not calculated due to this low variation among samples.

### 3.3 Microsatellite analysis

Of the 22 microsatellite markers, 19 were polymorphic in the Icelandic killer whale population (Appendix S1). The mean proportion of loci typed was 0.98 (nine samples failed to amplify at one locus, one at six loci and one at seven loci). All 61 genotypes were unique (the majority of the genotypes mismatched in at least three loci and only one pair of individual genotypes differed by one locus, possibly due to one of the genotypes lacking 7 out of 22 loci for matching purposes). Replicated samples showed no errors of re‐amplification or genotype. For all loci, MICRO‐CHECKER found no evidence for scoring errors or null alleles. When considering the whole dataset as a single population, five loci had significant departures from Hardy–Weinberg equilibrium after sequential Bonferroni correction. In all of these loci, there was higher observed heterozygosity than expected. However, when dividing the dataset into the genetic units identified by DAPC (see below), only two loci (FCB12 and TtruGT142) had significant departures after sequential Bonferroni correction in one genetic unit each (Appendix S2). As this deviation was not significant in all genetic units and results with and without these two loci were consistent (same number of genetic units and only two individual assignments changed), the loci were included in the analysis and only these results are reported. One pair of loci had significant linkage disequilibrium after sequential Bonferroni correction for multiple tests, but linkage disequilibrium was not detected when dividing the dataset into the DAPC genetic units and was therefore considered negligible.

### 3.4 Genetic differentiation and population subdivision

**Figure 2:**
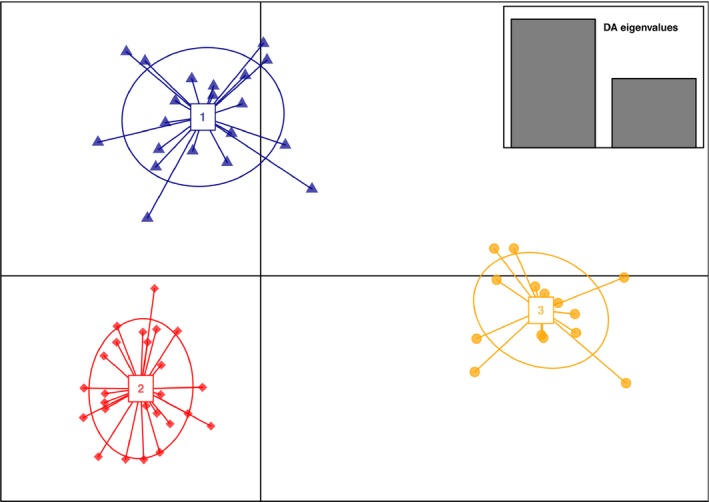
DAPC scatterplot showing the first two principal components for *K* = 3 (Genetic unit 1 = 1, Genetic unit 2 = 2, Genetic unit 3 = 3). Discriminant analysis (DA) eigenvalues are displayed in the inset

The probability support produced by STRUCTURE was highest for *K* = 1, indicating no subdivision among samples (see Appendix S3). However, three genetic units were identified by DAPC (Figure 2, see BIC plot in Appendix S4). The first component separated genetic unit 3 (*N* = 15, 12 males and three females) from genetic units 1 (*N* = 21, 14 males and seven females) and 2 (*N* = 25, 17 males and eight females), which were further separated by the second component (Figure 2). The removal of one individual per pair of closely related individuals (two and four individuals were removed from genetic units 1 and 2, respectively) did not change the inferred genetic units and individual memberships. Though the results with STRUCTURE indicate weak or no genetic structure, the DAPC approach was deemed appropriate to illustrate genetic structure patterns in this dataset (see Discussion section). Thus, the genetic units identified by DAPC were used in the subsequent analyses.

**Figure 3:**
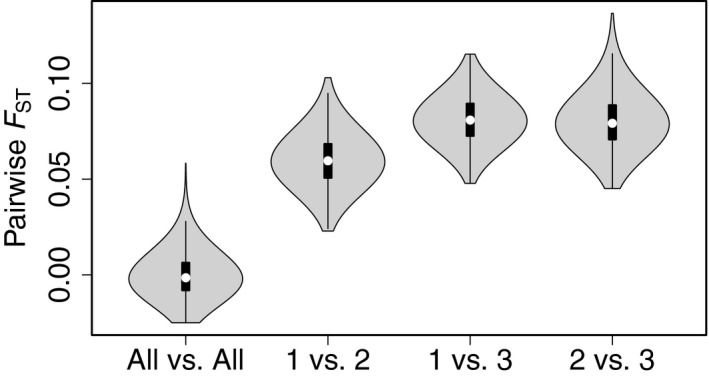
Violin plots of the randomized (1,000 iterations) microsatellite pairwise *F*_ST_ values between two groups of randomly selected individuals from: the whole dataset (All vs. All), genetic units 1 and 2 (1 vs. 2), genetic units 1 and 3 (1 vs. 3) and genetic units 2 and 3 (2 vs. 3). For each group of randomly selected individuals *N* = 10. The black central bar indicates the interquartile range and the white circles indicate the median value

Overall, microsatellite *F*_ST_ value (*F*_ST_ = 0.078, 95% CI: 0.039–0.130) and all pairwise *F*_ST _values were low but significant (genetic units 1 vs. 2 *F*_ST_ = 0.06; genetic units 1 vs. 3 and 2 vs. 3 *F*_ST_ = 0.09, *p*‐value < 0.001 for all pairwise *F*_ST_ values). Results were consistent when excluding closely related individuals. The randomization procedure confirmed that the pairwise *F*_ST_ values obtained were significantly greater than by sampling at random, with significant differences between the distribution of *F*_ST_ values of randomly selected individuals and the distribution of *F*_ST_ values for each pairwise comparison of the genetic units (Figure 3; *p*‐value < 0.001).

All genetic units showed high levels of observed heterozygosity (*H*_o_) in relation to expected values, and low number of alleles per locus (*k*) and allelic richness (AR) (Table 1, see Appendix S2 for values per loci). No comparisons of mean AR and *H*_o_ values among genetic units were significant, except for the significantly lower mean *H*_o_ in genetic unit 2 than in genetic unit 3 (Wilcoxon test, *p*‐value < 0.01; Table 1). Low numbers of private alleles were identified in genetic units 2 and 3 (Table 1). The mtDNA haplotypes were not completely discriminated but the majority of individuals in genetic units 1 and 2 had haplotype 33 while almost all individuals from genetic 3 had haplotype 34 (Table 1). This suggests a relationship between genetic unit and mtDNA haplotype (Fisher's Exact Test, *p*‐value < 0.0001).

**Table 1:** Number of mitochondrial haplotypes and microsatellite diversity, regarding the 19 polymorphic microsatellites, of Icelandic killer whales across the genetic units. Standard deviation shown in parenthesis

Genetic units	Mitochondrial haplotype		Microsatellites						
	33	34	*n*	*k*	AR	PA	*H*_o_	*H*_e_	*F*_IS_
1 (*N* = 21)	15	6	20.53 (0.77)	3.37 (1.54)	3.22 (1.33)	0	0.57 (0.32)	0.46 (0.23)	−0.25
2 (*N* = 25)	24	1	24.32 (1.11)	3.37 (1.83)	3.12 (1.54)	5	0.53 (0.32)	0.44 (0.22)	−0.21
3 (*N* = 15)	1	14	15 (0)	3.42 (1.17)	3.42 (1.17)	4	0.67 (0.25)	0.54 (0.18)	−0.24

Note

*n*: mean sample size per locus; *k*: mean number of alleles per locus; AR: mean allelic richness; PA: total number of private alleles; *H*_o_: mean observed heterozygosity; *H*_e_: mean expected heterozygosity; *F*_IS_: inbreeding coefficient.

### 3.5 Genetic units and movement patterns, isotopic niche width and social segregation

Genetic unit 1 included 14 individuals seen year‐round in Iceland and seven only seen seasonally (six in the summer and one in the winter). Genetic unit 2 included 16 individuals seen year‐round in Iceland and nine only seen seasonally (seven in the winter and two in the summer). Genetic unit 3 was composed of 14 individuals seen seasonally (nine in the winter and five in the summer) and only one individual seen in Iceland year‐round. This unit included all six individuals that are known to travel to Scotland. The difference in the ratio of individuals seen year‐round to individuals seen seasonally was significantly different among units (Fisher's Exact Test, *p*‐value < 0.001), with almost all of the individuals seen year‐round assigned to genetic units 1 and 2 (30/31 of the individuals seen year‐round).

**Figure 4:**
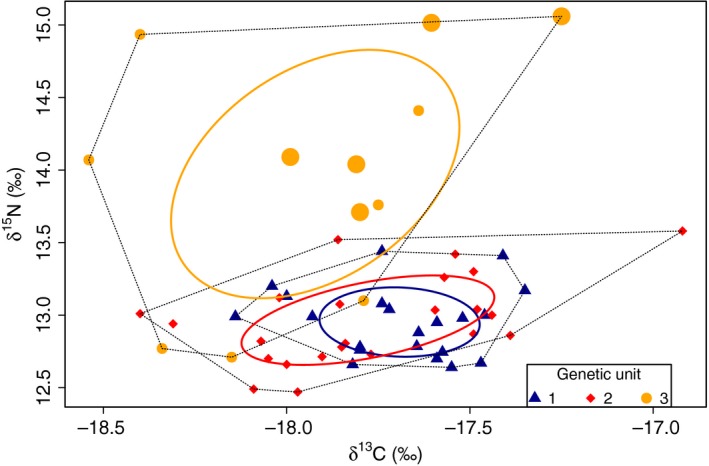
Isotopic values (δ^13^C and δ^15^N) of killer whales from each of the three genetic units. Solid lines represent the standard ellipses corrected for sample size (SEA_C_), while dashed lines represent the convex hull area. The five individuals from genetic unit 3 known to travel to Scotland for which there were isotopic data available are represented with larger circles

**Figure 5:**
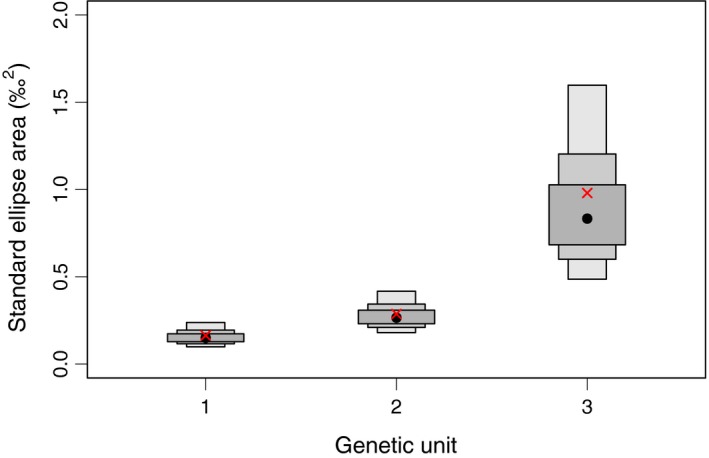
Distribution of the estimates of standard ellipse area (‰^2^) based on 2,000,000 iterations for killer whales belonging to the three genetic units inferred by DAPC. The black dots represent the mode, the red crosses represent the area of the standard ellipse corrected for sample size (SEAc) of the real data, and the shaded boxes represent the 50%, 75% and 95% credible intervals (from dark to light gray, respectively)

Stable isotope ratios were available for all 21 individuals of genetic unit 1, for 23/25 individuals of genetic unit 2 and for 12/15 individuals of genetic unit 3, including 5/6 individuals matched to Scotland in the summer (Samarra, Vighi, et al., 2017). Overall, individuals from genetic unit 3 had higher values of δ^15^N (Figure 4) and a significantly larger SEA_C_ than individuals from the two other genetic units (Figure 5; unit 3 vs. unit 1: 0.98 vs. 0.16‰^2^, *p*‐value < 0.0001; unit 3 vs. unit 2: 0.98 vs. 0.29‰^2^, *p*‐value < 0.001). The SEA_C_ of genetic units 1 and 2 largely overlapped (Figure 4) at the lower values of δ^15^N, although the SEA_C_ for unit 2 was slightly larger than unit 1 (*p*‐value = 0.03), particularly in the distribution of δ^13^C values.

**Figure 6:**
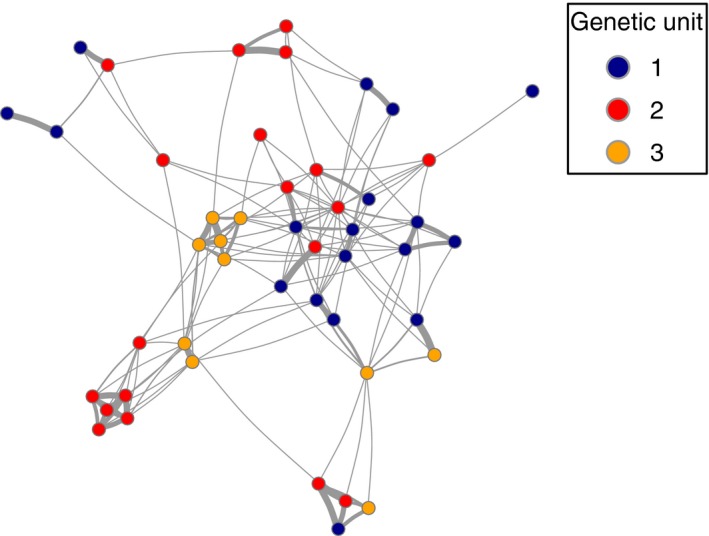
Social associations among killer whales from each of the three genetic units. Nodes represent individuals and edges (links) represent existing associations between individuals. Thicker edges correspond to higher half‐weight index (HWI) values. Network was plotted using Fruchterman‐Reingold force‐directed layout (Fruchterman & Reingold, 1991)

Association data were available for 18/21 individuals of genetic unit 1, for 19/25 individuals of genetic unit 2 and for 10/15 individuals of genetic unit 3 (Tavares et al., 2017). Considering the social clusters defined by Tavares et al. (2017), this comprised nine different social clusters in genetic unit 1, 11 in genetic unit 2 and five in genetic unit 3. Mean HWI within genetic units was significantly higher than among genetic units (mean ± standard deviation HWI_within_ vs. HWI_between_: 0.06 ± 0.16 vs. 0.02 ± 0.07, real difference of means = 0.04, 95% CI bootstrapped difference of means: −0.01 to 0.02, *p*‐value < 0.0001). However, there were still several associations among individuals from different genetic units (Figure 6; mean non‐zero HWI ± SD between genetic units 1 and 2 = 0.16 ± 0.22, units 1 and 3 = 0.16 ± 0.21, and units 2 and 3 = 0.10 ± 0.08) and some were strong (maximum HWI of 0.77, 0.86, and 0.33 between genetic units 1 and 2, 1 and 3 and 2 and 3, respectively), indicating recurrent associations between those individuals. It should be noted that, of the social clusters defined by Tavares et al. (2017) with individuals sampled in this study, six social clusters had members assigned to both genetic units 1 and 2, three social clusters had members assigned to both genetic units 1 and 3, four had members assigned to units 2 and 3, and two social clusters had members assigned to all of the three genetic units.

### 3.6 Sex‐biased dispersal, recent migration rates and changes in effective size

There was no significant sex‐biased dispersal across observed movement patterns detected in FSTAT, since neither sex‐specific *F*_ST_ nor vAI values were significantly different between males and females (Table 2). Individual *F*_IS_ values of each genetic unit were negative (Table 1), so no evidence of inbreeding was detected within the genetic units, suggesting outbreeding and gene flow with a genetically distinct population/subpopulation. Among genetic units, there was a relatively high number of effective migrants after correction for size (*N*_m_) of 4.75 migrants per generation, indicating gene flow among the genetic units. Recent migration rates estimated by BAYESASS were highly consistent among runs. High rates of recent gene flow were detected between genetic units 1 and 2 (30% of individuals per generation in genetic unit 1 were estimated to originate from genetic unit 2%, and 31% vice versa). In contrast, very low rates were detected between genetic units 1 and 3 (<2% of individuals originating between units per generation). Unidirectional gene flow was detected from genetic unit 2 into genetic unit 3 (29% of individuals per generation, with only 1% of unit 2 individuals estimated to have originated from unit 3). However, we infer these rates may not correspond to contemporary gene flow, as all individuals within each genetic unit had a high likelihood (≧88%) of being at least a 2^nd^ generation migrant.

**Table 2:** Sex‐biased dispersal test among genetic units. Differences in sex‐specific *F*_ST_ values and variance of corrected assignment index were tested for significance using 10,000 permutations

	*F*_ST_	vAI
Males	0.08	10.04
Females	0.09	9.23
*p*‐value	0.53	0.82

Note

vAI: variance of corrected assignment index.

BOTTLENECK showed marginal detection of recent changes in effective size for genetic units 2 and 3. Under the infinite alleles model there was a significant heterozygosity excess (genetic unit 2: *p*‐value = 0.005; genetic unit 3: *p*‐value = 0.001), but this was not verified under the two‐phase model (genetic unit 2: *p*‐value = 0.32; genetic unit 3: *p*‐value = 0.04 ‐ not significant after Bonferroni correction for multiple tests). Furthermore, the allele frequency distribution had a normal L‐shape for both genetic units, which is not expected if a recent bottleneck has occurred (Luikart, Allendorf, Cornuet, & Sherwin, 1998). Genetic unit 1 showed no significant indications of recent change in effective size (infinite alleles model: *p*‐value = 0.04; two‐phase model: *p*‐value = 0.41; normal L‐shaped allele frequency distribution).

## 4 DISCUSSION

### 4.1 Fine‐scale genetic structure

Our study supports weak fine‐scale genetic differentiation among Icelandic killer whales into three genetic units. Population subdivision was supported by DAPC but not by STRUCTURE. STRUCTURE uses a model based‐method that assumes Hardy–Weinberg equilibrium, which includes the strict assumption that populations are panmictic, discrete and reproductively isolated (Palsbøll, Peery, & Bérubé, 2010; Pritchard et al., 2000). When the assumptions are not met, the STRUCTURE analysis loses power to detect genetic structure. Furthermore, STRUCTURE can fail to identify genetic structure when: (a) allele frequencies vary gradually across a region (e.g., when there is isolation‐by‐distance) (Pritchard, Wen, & Falush, 2009); (b) sample size of genetic groups is limited or uneven (Kalinowski, 2011; Puechmaille, 2016; Waples & Gaggiotti, 2006); (c) gene flow is relatively high (Waples & Gaggiotti, 2006); and (d) mutation is low and genetic differentiation is limited (Almojil, Cliff, & Spaet, 2018; Barr et al., 2008; Latch, Dharmarajan, Glaubitz, & Rhodes, 2006; Waples & Gaggiotti, 2006). Contrarily, in cases of weak genetic differentiation, principal component analysis has proven to be a sensitive tool in detecting fine‐scale structure (Novembre et al., 2008; O'Connor et al., 2015); for example, using DAPC, Almojil et al. (2018) found biologically meaningful genetic clusters where STRUCTURE failed to find any (as in this study). Therefore, we believe that for our dataset, DAPC was a more sensitive approach to accurately investigate fine‐scale genetic differences among samples.

Overall, genetic units differed in isotopic signatures, mtDNA haplotype frequencies and observed movement patterns of the majority of its members. Although individuals associated more within the genetic unit (a result that can be affected by sampling bias of social groups), genetic units were not socially segregated and it is unlikely that association patterns were the main drivers of the genetic discontinuity observed. Since different social groups, as defined by Tavares et al. (2017), were sampled in this study and members of the same social clusters were assigned to different genetic units, it also seems unlikely that the sampling of some social groups more than others created the genetic structure observed among the samples.

The strongest level of genetic differentiation was found between individuals from genetic unit 3 and individuals from genetic units 1 and 2. Genetic unit 3 included individuals with overall higher δ^15^N values suggesting a broad diet (as discussed by Samarra, Vighi, et al., 2017), while individuals from genetic units 1 and 2 had a significantly narrower isotopic niche width and overall lower δ^15^N values, consistent with a diet predominantly composed of herring (as discussed by Samarra, Vighi, et al., 2017). Genetic units 1 and 2 had a significant predominance of mtDNA haplotype 33 and the majority of its individuals were seen year‐round in Iceland. Contrarily, nearly all individuals in genetic unit 3 had mtDNA haplotype 34 and were seen only seasonally in Iceland; some of them are known to travel to Scotland where they were seen feeding upon marine mammals (Samarra & Foote, 2015; Samarra, Vighi, et al., 2017). It is important to acknowledge that since the time lapse during which the stable isotopic signal of a given diet remains in skin is only a few weeks (Browning, Dold, I‐Fan, & Worthy, 2014; Giménez, Ramírez, Almunia, Forero, & de Stephanis, 2016), individual isotopic signatures might not represent the entire diet patterns of individuals. For example, the three individuals from genetic unit 3 (one seen in both seasons and two seen only in the summer) with δ^15^N values <13.5‰, the threshold encompassing all putative herring‐specialists (Samarra, Vighi, et al., 2017), could still have a broad diet but they might have been feeding mainly on herring before they were sampled. However, it is also possible that not all individuals in genetic unit 3 have a broad diet and that there was insufficient data to further differentiate genetic unit 3 into separate subunits.

There was further significant genetic structure separating genetic units 1 and 2. Between these units, we found a small difference in the distribution of the δ^13^C values, suggesting some variation in geographical foraging area (Bearhop, Adams, Waldron, Fuller, & Macleod, 2004), and while very few individuals assigned to genetic unit 1 were only seen in the winter in West Iceland (1/14), very few individuals of genetic unit 2 were only seen in the summer in South Iceland (2/16). However, it should be noted that observed movement patterns used here are incomplete descriptions of each individual's ranging pattern. Individuals seen in only one season might still have been present in both seasons but were missed, they might follow the Icelandic herring stock year‐round to a different herring ground, since there are more overwintering‐ and spawning‐grounds than those sampled in this study (ICES, 2015; Jakobsson & Stefánsson, 1999), or might not follow the stock, seasonally moving to other locations.

Genetic diversity indices, specifically observed heterozygosity (*H*_o_) and allelic richness (AR), were overall similar among genetic units: high *H*_o_ relative to expected values and low allelic richness. When populations experience a recent reduction of effective size, allelic diversity is reduced faster than heterozygosity (Piry et al., 1999). However, our analysis found no convincing evidence of recent reduction in effective size of the genetic units. Other causes of excess of heterozygotes can be over‐dominant selection (i.e., heterozygotes advantage due to higher fitness than homozygotes), outbreeding with a genetically distinct population, the presence of closely related or inbred family groups, the underlying social organizational level below that of the population and the composition of samples drawn from the real population (Beebee & Rowe, 2008; Kalinowski et al., 2007; Milkman, 1975; Parreira & Chikhi, 2015). We did not detect inbreeding, and the negative *F*_IS_ values supported genetic unit exogamy (i.e., outbreeding) while a high number of migrants (*N*_m_) indicated high gene flow.

### 4.2 Low genetic divergence

Genetic divergence among killer whales in Iceland with different isotopic signatures and observed movement patterns was weak, as supported by low overall and pairwise *F*_ST_ values. The magnitude of pairwise *F*_ST_ values is lower than the pairwise *F*_ST_ values based on microsatellite DNA (>0.1) reported between Northeast Pacific mammal‐eating and resident populations (e.g., Barrett‐Lennard, 2000; Hoelzel et al., 2007, 2002 ; Parsons et al., 2013). The *F*_ST_ values in our study are of similar magnitude to those in several mammal populations, assessed at the level of social groups with philopatry and gene flow among groups (Storz, 1999). Low genetic divergence has been commonly found in studies of marine species (e.g., Almojil et al., 2018; Vignaud et al., 2014; Ward, Woodwark, & Skibinski, 1994). There was also extremely low mtDNA haplotype variation in our dataset, consistent with low worldwide mtDNA diversity patterns in killer whales (e.g., Hoelzel et al., 2007, 2002 ; Morin et al., 2010; Moura, Janse van Rensburg, et al., 2014).

The level of genetic structure within the Icelandic population suggests variation in gene flow among killer whales in Iceland. The lack of indications of sex‐biased dispersal and the find that individuals were likely to be at least 2^nd^ generation migrants of other genetic units support the idea that gene flow is caused by other mechanism than differential dispersal of males or females and permanent migration of individuals among genetic units, respectively. Two main mechanisms could lead to the level of *F*_ST_ values observed in our study: (a) ongoing gene flow, with occasional outbreeding among genetic units, where the genetic differentiation is at an equilibrium state (less likely in view of the high likelihood of individuals being at least 2^nd^ generation migrants); or (b) recent historical separation of the genetic units, where the divergence is at non‐equilibrium state, and the gene flow among gene units is non‐existent or decreasing, but has not yet resulted in greater genetic differentiation. Little or non‐existent current gene flow would suggest that the current population has evolved rapidly from a historically more panmictic population. Additionally, these mechanisms could be influenced by several processes, such as: (a) differing rates of outbreeding with other North Atlantic populations; (b) preferential mating within subgroups; (c) isolation‐by‐distance/time/adaptation; and/or (d) periodic changes in connectivity among demes due to decadal changes in herring stock migration routes and separation into different herring feeding, spawning and wintering grounds.

### 4.3 Adaptive divergence and stability of genetic structure

Delphinid population structure is likely driven by a combination of socioecological processes (e.g., Amaral et al., 2012; Fontaine et al., 2007; Gaspari, Azzellino, Airoldi, & Hoelzel, 2007; Louis et al., 2014; Möller, 2012; Moura et al., 2015, 2013 ). Ecological variation and resource specialization are key factors driving divergence in killer whale populations (Foote et al., 2016, 2011 ; Hoelzel et al., 1998). From our results, it is possible to speculate that the first‐level of genetic division, separating genetic unit 3 from genetic units 1 and 2, corresponds to two main types of units: (a) individuals that potentially have a generalist diet and; (b) herring‐specialists. Furthermore, the second‐level of differentiation, separating genetic units 1 and 2, could be related to site‐fidelity to herring grounds and subsequent distribution of the individuals along the coast of Iceland. For example, the genetic structure of southern right whales (*Eubalaena australis*) is maintained since site‐fidelity to feeding areas is transmitted along matrilineal lines (Carroll et al., 2015; Valenzuela et al., 2009). However, in this speculative scenario, genetic divergence has not led to ecotypes as seen in the Northeast Pacific. First, the Icelandic genetic units seem substantially less genetically differentiated, as indicated by microsatellite DNA differentiation, and less ecologically discrete, as indicated by isotopic values and apparent prey overlap. Second, while Northeast Pacific killer whale ecotypes do not have mtDNA haplotypes in common (Barrett‐Lennard, 2000; Hoelzel et al., 2007, 2002), the Icelandic genetic units shared mtDNA haplotypes. Third, while between the mammal‐eating and resident ecotypes and between resident subpopulations there is strong social avoidance (e.g., Ford et al., 1998; Ford et al., 2000), killer whales in Iceland assigned to different genetic units were not socially isolated and engaged in associations at least occasionally (Tavares et al., 2017). Our results suggest that if killer whales in Iceland are progressing toward ecological divergence and niche specialization, this process is still at a very early stage. However, further exploration of different ecological niches by the different genetic units within the Icelandic population could eventually lead to adaptive variation and the formation of different ecotypes if it took place with stronger geographic and/or genetic isolation.

Our study raises the question of whether the fine‐scale genetic structure observed among Icelandic killer whales is stable long‐term and driven by local adaptation (e.g., through adaptation of foraging strategies for resource exploitation) or a subtle temporal structure driven by the contemporary distribution of fragmented seasonal herring grounds. Geographic overlap in Icelandic herring grounds and the fluidity of the feeding aggregations (Tavares et al., 2017) may create a unique social opportunity that promotes gene flow between individuals from different genetic units, at least in seasonal herring grounds. Gene flow dependent on seasonal spatiotemporal overlap of non‐dispersing individuals is a pattern of mating seen within the Northeast Pacific ecotypes (e.g., Hoelzel et al., 2007; Parsons et al., 2013; Pilot, Dahlheim, & Hoelzel, 2010) and other low‐dispersal species (e.g., long‐finned pilot whales *Globicephala melas*, Amos, Schlötterer, & Tautz, 1993; African elephant *Loxodonta africana*, Nyakaana & Arctander, 1999). In this scenario, gene flow is not possible between individuals that never spatially overlap, for example, individuals that visit herring grounds at different times during the season or visit different herring grounds. Furthermore, herring is a prey known to change migration routes and abundance (Jakobsson & Stefánsson, 1999; Óskarsson, Gudmundsdottir, & Sigurdsson, 2009). Future changes in herring distribution might influence the spatiotemporal overlap or the level of dispersal from Iceland of different individuals and, consequently, the opportunities for social interactions and genetic admixture.

Metapopulation dynamics can arise when patches of a fragmented habitat are momentarily occupied and are followed by local extinction when conditions become unsuitable and then reoccupied again sometime later (Beebee & Rowe, 2008; McQuinn, 1997). Continuous variation in genetic differentiation due to metapopulation dynamics would agree with the observed evolutionary patterns of several niche diversifications along genealogical lines of North Atlantic killer whales across thousands of years (Foote et al., 2013). Additionally, metapopulation dynamics and local adaptation are not mutually exclusive and can simultaneously influence the structure of a population. For example, the sampling over consecutive years at multiple within‐river sites of Atlantic salmon *(Salmo salar)* in the Sainte‐Marguerite river (Canada) showed that the population had significant substructuring in both space and time (Garant et al., 2000). It is possible that a similar scenario is driving the patterns of differentiation at a relatively fine‐scale within the Icelandic population. In such a situation, changes in movement patterns and geographic overlap could lead to future changes in the level of genetic divergence among Icelandic killer whales. Further studies investigating genetic differentiation at different time and space scales are needed to fully understand genetic structure among killer whales in Icelandic waters.

## CONFLICT OF INTEREST

None declared.

## AUTHOR CONTRIBUTIONS

S.B.T., F.I.P.S., and P.J.O.M. conceptualized the work and S.B.T., S.P., and J.A.G. conceptualized the analyses. S.B.T., F.I.P.S., and P.J.O.M. collected samples. S.B.T. performed laboratory work. S.B.T., S.P., and J.A.G. analyzed data. S.B.T. wrote the paper. All authors approved the final manuscript.

## DATA ACCESSIBILITY

Mitochondrial haplotypes and microsatellite genotypes used in this study are available from the Dryad Digital Repository: https://doi.org/10.5061/dryad.674k8j4.
