# Supplementary material for: Killer whales (Orcinus orca) in Iceland show weak genetic structure among diverse isotopic signatures and observed movement patterns
Source: Ecol Evol. 2018 Nov 14;8(23):11900–13. doi: 10.1002/ece3.4646 (PMC6303705; doi:10.1002/ece3.4646)
Supplement: Supplementary file 1 [file ECE3-8-11900-s001.docx]

**Supplemental Information for:**

**Killer whales (*Orcinus orca*) in Iceland show weak genetic structure among diverse isotopic signatures and observed movement patterns**

Sara B. Tavares, Filipa I. P. Samarra, Sonia Pascoal, Jeff A. Graves, Patrick J.O. Miller

**Table of Contents:**

| **Appendix S1** | Page 2 |
| --- | --- |
| **Appendix S2** | Page 4 |
| **Appendix S3** | Page 7 |
| **Appendix S4** | Page 8 |

Appendix S1. PCR, genotyping conditions and characteristics of each microsatellite locus. Ta is the annealing temperature. F is the forward primer sequence. R is the reverse primer sequence. The 22 loci were tested and their optimum PCR conditions determined in a small number of samples. For this, each 10 μl PCR contained 1 μl of 10-20 ng of extracted DNA, 1x PCR buffer, 1.5 mM MgCl_2_, 0.2 μM of each primer, 0.2 mM of mixed dNTPs and 0.05 μl of AmpliTaq® DNA Polymerase (Applied Biosystems, Foster City, CA, USA). PCR amplifications were performed using a G-Storm GS1 thermal cycler (Gene Technologies) with an initial denaturation step at 95 ºC for 2 min, followed by the specific number of cycles for each loci of denaturation at 95 ºC for 15 sec, annealing for 30 sec (except for 464/465 and MK5 which was for 60 and 45 sec, respectively) at the specific temperature for each loci and extension at 72ºC for 1 min, followed by a final extension at 72 ºC for 5 min. Successful amplification was confirmed using agarose gel, ethidium bromide (EtBr) staining and UV visualisation.

|  |  |  |  |  | Optimal PCR conditions | | Multiplex PCR | |
| --- | --- | --- | --- | --- | --- | --- | --- | --- |
| Locus | Reference | Size range (bp) | Variability ^a^ | Primer sequence (5’-3’) | Number of cycles | T_a_ (°C) | Label | Mix |
| 464/465 ^c^ | (Schlötterer, Amos, & Tautz, 1991) | 121-123 | P | F: TCTCCTCTAAATTCATGCCCC  R: AGGCATGACTTACAGAGGAGT | 35 | 56 | FAM | I |
| MK5 ^b, c^ | (Krützen, Valsecchi, Connor, & Sherwin, 2002) | 238-242 | P | F: CTCAGAGGGAAATGAGGCTG  R: TGTCTAGAGGTCAAAGCCTTCC | 35 | 64 | PET | I |
| KWM12a ^c^ | (Hoelzel, Dahlheim, & Stern, 1998) | 192-200 | P | F: CCATACAATCCAGCAGTC  R: CACTGCAGAATGATGACC | 30 | 60 | VIC | I |
| Dde72 ^c^ | (Coughlan, Mirimin, Dillane, Rogan, & Cross, 2006) | 288-296 | P | F: TGCTCAACAGATTTCACACTT  R: AAGGAAACAAAGTATCTGAGCA | 33 | 61 | NED | I |
| Dde66 ^c^ | (Coughlan et al., 2006) | 374-386 | P | F: AACATTGCCAGTGCCTTAGAA  R: GTGGAACAGACGCGCATAT | 33 | 64 | FAM | I |
| Dde70 ^c^ | (Coughlan et al., 2006) | 149-157 | P | F: ACACCAGCACCTACATTCACA  R: TCAGCAGCATTCTAACCAAAC | 33 | 55 | FAM | II |
| KW4 ^c^ | (Ford et al., 2011) | 200-204 | P | F: AAATAGCTCAGGGGCCAGAC  R: AGCTGGAGTTGTTTGCTGTG | 33 | 60 | PET | II |
| FCB4 ^b^ | (Buchanan, Friesen, Littlejohn, & Clayton, 1996) | 222-262 | P | F: CCTGTCAGGAGAATTGAGGTATCC  R: GGATAAGGCCATTAGCCTCCACC | 33 | 55 | VIC | II |
| TtruGT48 ^c^ | (Caldwell, Gaines, & Hughes, 2002) | 193-215 | P | F: GAGAAAAGAAAACTCTGCCTGAA  R: CCAGGACTTCCCCCAATACT | 33 | 55 | NED | II |
| BA417 ^b^ | (Hoelzel et al. 1998, after Amos, Schlötterer, & Tautz, 1993) | 190 | M | F: TACAGTATTTGTCTTTCTCT  R: ATCTGTTTGTCACATATCAT | 33 | 54 | FAM | II |
| D22 ^b^ | (Shinohara, Domingo-Roura, & Takenaka, 1997) | 144-154 | P | F: GGAAATGCTCTGAGAAGGTC  R: CCAGAGCACCTATGTGGAC | 33 | 54 | VIC | III |
| FCB17 ^b, c^ | (Buchanan et al., 1996) | 172-174 | P | F: TCAGCCTCTATAACGTCCTGAGC  R: ATGGGGACTGCCTATATTAGTCAG | 33 | 54 | NED | III |
| EV37 ^b, c^ | (Valsecchi & Amos, 1996) | 215-227 | P | F: AGCTTGATTTGGAAGTCATGA  R: TAGTAGAGCCGTGATAAAGTGC | 33 | 54 | FAM | III |
| Ttr04 ^b, c^ | (Rosel, Forgetta, & Dewar, 2005) | 124-128 | P | F: CTGACCAGGCACTTTCCAC  R: GTTTGTTTCCCAGGATTTTAGTGC | 33 | 54 | FAM | IV |
| TtruGT142 ^b, c^ | (Caldwell et al., 2002) | 218-230 | P | F: CTGGGTCAAAAAGGAAGAGC  R: CCGCTGGGAAGAAACAATAG | 33 | 57 | PET | IV |
| TtruAAT44 ^b^ | (Caldwell et al., 2002) | 119-122 | P | F: CCTGCTCTTCATCCCTCACTAA  R: CGAAGCACCAAACAAGTCATAGA | 33 | 54 | VIC | IV |
| FCB5 ^b, c^ | (Buchanan et al., 1996) | 149-163 | P | F: CTCCTCATGGTCAGACTCCCAG  R: GTACATTTACCCATTCAGAACTTTGG | 33 | 54 | NED | IV |
| FCB12 ^b, c^ | (Buchanan et al., 1996) | 190-194 | P | F: CTCAGTTAATATACATGTAATGCATGC  R: CAAAGAGATAGCTAAATAAACAGTAAC | 33 | 48 | FAM | IV |
| KW2a ^b^ | (Hoelzel et al., 1998) | 169 | M | F: GCTGTGAAAATTAAATGT  R: CACTGTGGACAAATGTAA | 33 | 46 | FAM | V |
| EV1 ^b, c^ | (Valsecchi & Amos, 1996) | 143-151 | P | F: CCCTGCTCCCCATTCTC  R: ATAAACTCTAATACACTTCCTCCAAC | 33 | 51 | VIC | V |
| D08 ^b^ | (Shinohara et al., 1997) | 105 | M | F: GATCCATCATATTGTCAAGTT  R: TCCTGGGTGATGAGTCTTC | 33 | 54 | NED | V |
| Ttr11 ^b, c^ | (Rosel et al., 2005) | 229-237 | P | F: CTTTCAACCTGGCCTTTCTG  R: GTTTGGCCACTACAAGGGAGTGAA | 33 | 50 | FAM | V |

^a^ P = polymorphic (>1 different allele), M = monomorphic (1 allele size).

^b^ Used in previous study of genetic differentiation among North Atlantic killer whales (Foote et al., 2011).

^c^ Used in previous study of genetic differentiation among northern North Pacific killer whales (Parsons et al., 2013).

References

Amos, B., Schlötterer, C., & Tautz, D. (1993). Social structure of pilot whales revealed by analytical DNA profiling. *Science*, *260*(5108), 670–672.

Buchanan, F. C., Friesen, M. K., Littlejohn, R. P., & Clayton, J. W. (1996). Microsatellites from the beluga whale Delphinapterus leucas. *Molecular Ecology*, *5*(4), 571–575.

Caldwell, M., Gaines, M. S., & Hughes, C. R. (2002). Eight polymorphic microsatellite loci for bottlenose dolphin and other cetacean species. *Molecular Ecology Notes*, *2*(4), 393–395.

Coughlan, J., Mirimin, L., Dillane, E., Rogan, E., & Cross, T. F. (2006). Isolation and characterization of novel microsatellite loci for the short-beaked common dolphin (Delphinus delphis) and cross-amplification in other cetacean species. *Molecular Ecology Notes*, *6*(2), 490–492.

Foote, A. D., Vilstrup, J. T., de Stephanis, R., Verborgh, P., Abel Nielsen, S. C., Deaville, R., … Piertney, S. B. (2011). Genetic differentiation among North Atlantic killer whale populations. *Molecular Ecology*, *20*(3), 629–641.

Ford, M. J., Hanson, M. B., Hempelmann, J. A., Ayres, K. L., Emmons, C. K., Schorr, G. S., … Balcomb-Bartok, K. (2011). Inferred paternity and male reproductive success in a killer whale (Orcinus orca) population. *Journal of Heredity*, *102*(5), 537–553.

Hoelzel, A. R., Dahlheim, M. E., & Stern, S. J. (1998). Low genetic variation among killer whales (Orcinus orca) in the eastern North Pacific and genetic differentiation between foraging specialists. *The Journal of Heredity*, *89*(2), 121–128.

Krützen, M., Valsecchi, E., Connor, R. C., & Sherwin, W. B. (2002). Characterization of microsatellite loci in Tursiops aduncus. *Molecular Ecology Notes*, *1*(3), 170–172.

Parsons, K. M., Durban, J. W., Burdin, A. M., Burkanov, V. N., Pitman, R. L., Barlow, J., … Wade, P. R. (2013). Geographic patterns of genetic differentiation among killer whales in the Northern North Pacific. *Journal of Heredity*, *104*(6), 737–754.

Rosel, P. E., Forgetta, V., & Dewar, K. (2005). Isolation and characterization of twelve polymorphic microsatellite markers in bottlenose dolphins (Tursiops truncatus). *Molecular Ecology Notes*, *5*(4), 830–833.

Schlötterer, C., Amos, B., & Tautz, D. (1991). Conservation of polymorphic simple sequence loci in cetacean species. *Nature*, *353*(6348), 63–65.

Shinohara, M., Domingo-Roura, X., & Takenaka, O. (1997). Microsatellites in the bottlenose dolphin Tursiops truncatus. *Molecular Ecology*, *6*(7), 695–696.

Valsecchi, E., & Amos, W. (1996). Microsatellite markers for the study of cetacean populations. *Molecular Ecology*, *5*(1), 151–156.

Appendix S2. Test for deviation from Hardy-Weinberg equilibrium (HWE) of each polymorphic locus in each genetic unit and in the whole dataset. *P*-values that are significant after sequential Bonferroni correction for multiple tests are highlighted in bold (*P_crit_* for most significant test for the whole dataset equals 0.0026 [k = 19] and *P_crit_* for most significant test for the genetic units equals 0.0009 [k = 57]). The mean sample size per locus (n), the mean number of alleles per locus (k), the mean allelic richness (AR), the number of private alleles (PA), the mean observed heterozygosity (H_o_), the mean expected heterozygosity (H_e_) and the inbreeding coefficient (F_IS_) were also calculated for each polymorphic locus in each genetic unit and in the whole dataset when appropriate.

| Dataset | Locus |  | HWE | n | k | AR | PA | H_o_ | H_e_ | F_IS_ |
| --- | --- | --- | --- | --- | --- | --- | --- | --- | --- | --- |
| Genetic unit 1 | 464/465 |  | 1.0000 | 21 | 2 | 1.92 |  | 0.10 | 0.09 | -0.03 |
|  | MK5 |  | 0.0136 | 21 | 3 | 3.00 |  | 0.91 | 0.61 | -0.51 |
|  | KWM12a |  | 0.0416 | 21 | 4 | 4.00 |  | 0.91 | 0.67 | -0.36 |
|  | Dde72 |  | 0.4741 | 21 | 3 | 3.00 |  | 0.71 | 0.62 | -0.15 |
|  | Dde66 |  | 0.2204 | 21 | 4 | 3.71 |  | 0.33 | 0.37 | 0.11 |
|  | Dde70 |  | 1.0000 | 18 | 3 | 3.00 |  | 0.72 | 0.66 | -0.11 |
|  | KW4 |  | 0.2897 | 21 | 3 | 3.00 |  | 0.43 | 0.46 | 0.07 |
|  | FCB4 |  | 0.3515 | 21 | 7 | 5.99 |  | 0.62 | 0.65 | 0.05 |
|  | TtruGT48 |  | 0.0097 | 21 | 6 | 5.62 |  | 1.00 | 0.72 | -0.40 |
|  | D22 |  | 0.7029 | 21 | 6 | 5.35 |  | 0.71 | 0.66 | -0.08 |
|  | FCB17 |  | 0.0215 | 21 | 2 | 2.00 |  | 0.71 | 0.47 | -0.54 |
|  | EV37 |  | 0.3445 | 20 | 4 | 4.00 |  | 0.75 | 0.62 | -0.21 |
|  | Ttr04 |  | 1.0000 | 20 | 2 | 2.00 |  | 0.20 | 0.19 | -0.09 |
|  | TtruGT142 |  | 0.0018 | 20 | 4 | 3.94 |  | 0.95 | 0.69 | -0.40 |
|  | TtruAAT44 |  | - | 20 | 2 | 1.75 |  | 0.05 | 0.05 | 0.00 |
|  | FCB5 |  | 0.7548 | 20 | 3 | 3.00 |  | 0.50 | 0.42 | -0.21 |
|  | FCB12 |  | **0.0006** | 20 | 2 | 2.00 |  | 0.90 | 0.51 | -0.81 |
|  | EV1 |  | 1.0000 | 21 | 2 | 2.00 |  | 0.29 | 0.25 | -0.14 |
|  | Ttr11 |  | 1.0000 | 21 | 2 | 1.92 |  | 0.10 | 0.09 | -0.03 |
| Genetic unit 2 | 464/465 |  | - | 25 | 1 | 1.00 |  | 0.00 | 0.00 | - |
|  | MK5 |  | 0.0709 | 25 | 3 | 3.00 |  | 0.80 | 0.59 | -0.37 |
|  | KWM12a |  | 0.0016 | 25 | 4 | 3.59 |  | 0.88 | 0.61 | -0.45 |
|  | Dde72 |  | 0.3850 | 25 | 3 | 2.60 |  | 0.20 | 0.25 | 0.21 |
|  | Dde66 |  | 0.1880 | 25 | 3 | 2.60 |  | 0.28 | 0.28 | 0.00 |
|  | Dde70 |  | 0.3800 | 23 | 4 | 3.88 | 1 | 0.83 | 0.63 | -0.32 |
|  | KW4 |  | 1.0000 | 25 | 2 | 2.00 |  | 0.24 | 0.22 | -0.12 |
|  | FCB4 |  | 0.6903 | 25 | 7 | 5.78 | 1 | 0.76 | 0.69 | -0.10 |
|  | TtruGT48 |  | 0.0594 | 25 | 8 | 6.95 | 2 | 1.00 | 0.75 | -0.34 |
|  | D22 |  | 0.0748 | 25 | 5 | 4.79 |  | 0.84 | 0.69 | -0.23 |
|  | FCB17 |  | 0.5433 | 25 | 2 | 2.00 |  | 0.40 | 0.33 | -0.23 |
|  | EV37 |  | 0.7119 | 21 | 4 | 3.92 |  | 0.67 | 0.56 | -0.19 |
|  | Ttr04 |  | 0.6357 | 24 | 2 | 2.00 |  | 0.46 | 0.40 | -0.14 |
|  | TtruGT142 |  | **<0.0001** | 23 | 4 | 3.99 |  | 1.00 | 0.67 | -0.51 |
|  | TtruAAT44 |  | - | 24 | 1 | 1.00 |  | 0.00 | 0.00 | - |
|  | FCB5 |  | 0.7307 | 24 | 4 | 3.49 | 1 | 0.50 | 0.47 | -0.06 |
|  | FCB12 |  | 0.6284 | 23 | 3 | 2.65 |  | 0.44 | 0.36 | -0.23 |
|  | EV1 |  | 0.2868 | 25 | 2 | 2.00 |  | 0.28 | 0.39 | 0.29 |
|  | Ttr11 |  | 1.0000 | 25 | 2 | 2.00 |  | 0.44 | 0.39 | -0.12 |
| Genetic unit 3 | 464/465 |  | 1.0000 | 15 | 2 | 2.00 |  | 0.20 | 0.19 | -0.08 |
|  | MK5 |  | 0.1848 | 15 | 3 | 3.00 |  | 0.87 | 0.67 | -0.30 |
|  | KWM12a |  | 0.0994 | 15 | 4 | 4.00 |  | 0.87 | 0.63 | -0.38 |
|  | Dde72 |  | 0.2654 | 15 | 3 | 3.00 |  | 0.73 | 0.55 | -0.35 |
|  | Dde66 |  | 0.5682 | 15 | 3 | 3.00 |  | 0.53 | 0.54 | 0.00 |
|  | Dde70 |  | 0.7836 | 15 | 4 | 4.00 | 1 | 0.73 | 0.70 | -0.05 |
|  | KW4 |  | 1.0000 | 15 | 3 | 3.00 |  | 0.33 | 0.30 | -0.13 |
|  | FCB4 |  | 0.8209 | 15 | 5 | 5.00 |  | 0.73 | 0.71 | -0.03 |
|  | TtruGT48 |  | 0.0399 | 15 | 6 | 6.00 | 1 | 1.00 | 0.71 | -0.43 |
|  | D22 |  | 0.3590 | 15 | 5 | 5.00 |  | 0.73 | 0.64 | -0.15 |
|  | FCB17 |  | 0.1418 | 15 | 2 | 2.00 |  | 0.73 | 0.52 | -0.44 |
|  | EV37 |  | 0.6392 | 15 | 5 | 5.00 | 1 | 0.93 | 0.74 | -0.27 |
|  | Ttr04 |  | 0.5969 | 15 | 3 | 3.00 | 1 | 0.33 | 0.38 | 0.14 |
|  | TtruGT142 |  | 0.0024 | 15 | 4 | 4.00 |  | 1.00 | 0.70 | -0.45 |
|  | TtruAAT44 |  | 1.0000 | 15 | 2 | 2.00 |  | 0.13 | 0.13 | -0.04 |
|  | FCB5 |  | 1.0000 | 15 | 3 | 3.00 |  | 0.73 | 0.61 | -0.22 |
|  | FCB12 |  | 0.3074 | 15 | 3 | 3.00 |  | 0.67 | 0.48 | -0.41 |
|  | EV1 |  | 0.1242 | 15 | 2 | 2.00 |  | 0.73 | 0.51 | -0.47 |
|  | Ttr11 |  | 0.3565 | 15 | 3 | 3.00 |  | 0.67 | 0.58 | -0.16 |
| Whole dataset | 464/465 |  | 1.0000 | 61 | 2 | 2.00 |  | 0.08 | 0.08 | -0.03 |
|  | **MK5** |  | **0.0003** | 61 | 3 | 3.00 |  | 0.85 | 0.62 | -0.38 |
|  | **KWM12a** |  | **<0.0001** | 61 | 4 | 4.00 |  | 0.89 | 0.64 | -0.39 |
|  | Dde72 |  | 0.5879 | 61 | 3 | 3.00 |  | 0.51 | 0.48 | -0.06 |
|  | Dde66 |  | 0.1307 | 61 | 4 | 3.99 |  | 0.36 | 0.40 | 0.09 |
|  | Dde70 |  | 0.2591 | 56 | 5 | 5.00 |  | 0.77 | 0.68 | -0.13 |
|  | KW4 |  | 0.0848 | 61 | 3 | 3.00 |  | 0.33 | 0.32 | -0.02 |
|  | FCB4 |  | 0.6051 | 61 | 8 | 7.92 |  | 0.71 | 0.69 | -0.03 |
|  | **TtruGT48** |  | **<0.0001** | 61 | 9 | 8.83 |  | 1.00 | 0.77 | -0.31 |
|  | D22 |  | 0.0138 | 61 | 6 | 6.00 |  | 0.77 | 0.67 | -0.16 |
|  | FCB17 |  | 0.0104 | 61 | 2 | 2.00 |  | 0.59 | 0.44 | -0.33 |
|  | EV37 |  | 0.0570 | 56 | 5 | 5.00 |  | 0.77 | 0.64 | -0.21 |
|  | Ttr04 |  | 1.0000 | 59 | 3 | 2.95 |  | 0.34 | 0.33 | -0.03 |
|  | **TtruGT142** |  | **<0.0001** | 58 | 4 | 4.00 |  | 0.98 | 0.70 | -0.42 |
|  | TtruAAT44 |  | 1.0000 | 59 | 2 | 2.00 |  | 0.05 | 0.05 | -0.02 |
|  | FCB5 |  | 0.2583 | 59 | 4 | 3.95 |  | 0.56 | 0.65 | 0.14 |
|  | **FCB12** |  | **<0.0002** | 58 | 3 | 3.00 |  | 0.66 | 0.46 | -0.45 |
|  | EV1 |  | 0.7579 | 61 | 2 | 2.00 |  | 0.39 | 0.42 | 0.06 |
|  | Ttr11 |  | 0.4495 | 61 | 3 | 3.00 |  | 0.38 | 0.40 | 0.05 |

Appendix S3. (A) Assignment probabilities of individual killer whales inferred by STRUCTURE for K=2 plotted using POPHELPER v1.0.10 (Francis 2016): each vertical column corresponds to one individual, with the colours representing the membership proportion to each of the two clusters. White dotted line delimits the DAPC genetic units (1 – Genetic unit 1; 2 – Genetic unit 2; 3 – Genetic unit 3). (B) Results from the STRUCTURE analysis for K = 1-10 plotted using STRUCTURE HARVESTER v.0.6.94 (Earl and VonHoldt 2012): Mean log likelihood [L(K)] (bottom left) and Evanno plot of second order rate of change constant (∆K) (bottom right). The most likely number of clusters identified in STRUCTURE using the Evanno method (Evanno et al. 2005) was two (K = 2; see figure above), with one cluster composed of 32 individuals and the other of 29 individuals. Considering the results from STRUCTURE for K = 2 and the division into two main groups by the first principal component of DAPC (genetic units 1 and 2 versus genetic unit 3), assignments were somewhat consistent among the methods, with 77% of the individuals assigned to the same group. However, the ∆K method of Evanno et al. (2005) cannot discriminate K = 1 from K = 2 and the mean L(K) of K = 1 was slightly higher. Furthermore, only 49% of the individuals were strongly assigned to one of the clusters at K = 2 (assignment probability >0.80).

References

Earl, D. A., & vonHoldt, B. M. (2012). STRUCTURE HARVESTER: a website and program for visualizing STRUCTURE output and implementing the Evanno method. Conservation Genetics Resources, 4(2), 359–361.

Evanno, G., Regnaut, S., & Goudet, J. (2005). Detecting the number of clusters of individuals using the software structure: a simulation study. Molecular Ecology, 14(8), 2611–2620.

Francis, R. M. (2016). pophelper: an r package and web app to analyse and visualize population structure. Molecular Ecology Resources, 27–32.

Appendix S4. Selection of the optimal number of clusters (K = 3) for the DAPC analysis using the lowest BIC (Bayesian Information Criterion).
